# Detecting thin adhesive coatings in wood fiber materials with laboratory-based dual-energy computed tomography (DECT)

**DOI:** 10.1038/s41598-022-20422-1

**Published:** 2022-09-24

**Authors:** Pierre Kibleur, Benjamin Blykers, Matthieu N. Boone, Luc Van Hoorebeke, Joris Van Acker, Jan Van den Bulcke

**Affiliations:** 1 UGent-Woodlab, Department of Environment, Faculty of Bioscience Engineering, Ghent University, Coupure Links 653, 9000 Ghent, Belgium; 2 PProGRess, Department of Geology, Faculty of Sciences, Ghent University, Krijgslaan 281, S8, 9000 Ghent, Belgium; 3 Radiation Physics, Department of Physics and astronomy, Faculty of Sciences, Ghent University, Proeftuinstraat 86, 9000 Ghent, Belgium; 4 Ghent University Center for X-ray Tomography (UGCT), Proeftuinstraat 86, 9000 Ghent, Belgium

**Keywords:** Imaging techniques, Composites

## Abstract

The distribution and good spreading of adhesive resins is critical for the wood-based panels industry. Full 3D non-destructive characterization is necessary, but methods are limited due to the chemical similarities between the resins and the wood fibers. For X-ray microtomography ($\mu$CT), the doping of the resin with a highly attenuating contrast agent is necessary to visualize the resin distribution. However, the attenuation signal remains hard to segment clearly due to partial volume effects in the image, and phase mixing in the material. To help in the identification of the doped resin, dual-energy X-ray CT (DECT) is used to exploit the contrast agent’s K-edge, based on simulations which take into account the polychromatic properties of the X-ray tube and detector response. The contrast agent’s identification with DECT is validated with elemental mapping using scanning electron microscopy combined with energy-dispersive spectroscopy (SEM-EDX) on the surface of a wood-based panel sample, using data fusion between DECT and SEM-EDX. Overall, DECT results here in the first 3D identification of doped resin inside wood fiberboards, guiding the industry’s efforts in further improving the durability of wood-based panels.

## Introduction

Wood-based panels (WBPs) are engineered wood products consisting of different grades of wood pieces that are bound into homogeneous construction materials. When the wood pieces that compose the panel present consistently large and flat surfaces (e.g. in plywood or cross-laminated timber), the binding is done with locally-applied adhesive resins. When the wood pieces are smaller (e.g. flake-, fiber- or particle-boards), adhesives are globally sprayed on the wood pieces, but the enormous surface area to cover makes it difficult to obtain an ideal spread. As the most expensive component by weight, and up to 32% of the total manufacturing cost in WBPs^1^, reducing the adhesive content and optimizing its distribution in WBPs are of major interest to manufacturers. They have a strong incentive to reduce the adhesive amounts without altering the panel’s properties. Apart from production costs, finer resin distribution instead of coarse adhesive droplets improves the panel’s mechanical properties^1^. Reducing the amount of adhesive resin by reaching a better spread can also increase the recyclability of WBPs, which is currently still low^2^. The most common type of adhesive in the WBP industry is urea-formaldehyde (UF) resin (>90 % of WBPs)^3,4^.

Medium-density fiberboard (MDF) is one of the three principal WBPs. It consists of a network of wood fibers with in-plane isotropic orientation, mixed with an adhesive resin. The wood fibers, which interact with the UF resin in MDF, are hollow tubular structures composed of a wood cell wall surrounding an inner void called the lumen. Pores and micropores in the cell wall help with the adhesive penetration, which is expected to reach around 50% of the wall’s thickness^4^. While glue penetration is necessary to hold the fibers together, excessive penetration is a waste of resin^4^. The binding between the fiber and the adhesive is helped by the compatibility between the polar groups found in both the wood and the UF resin^5^. Nonetheless, resin tends to accumulate around small wood particles (highest surface-to-volume ratio), which absorb most of it without providing much fiber binding^1^. Although they could not be imaged inside a real sample yet, localized glue points are expected to form in MDF, instead of a continuum^6^. Glue points, and the heterogeneity of resin distribution could be observed with digital microscopy before hot pressing^7^, or investigated with confocal microscopy^4,8^. Other methods include near-infrared spectroscopy, or fluorescence X-ray spectroscopy^9^. However, these techniques only allow to image a few wood fibers extracted from MDF or put aside before pressing, where the 3D imaging of a piece of an MDF panel is necessary to investigate the resin distribution within the panel.

X-ray microtomography ($\mu$CT) allows for high-resolution, 3D and non-destructive imaging of MDF, but is weak to identify chemicals. Its signal depends on material density and atomic number^10^. When these are too similar, no distinction can be made between a material’s constituents from one voxel (a 3D pixel) to the next. In wood science, the elemental compositions of the constituents are mainly C and O, in comparable proportions throughout. Therefore, $\mu$CT can be considered to yield a 3D map of density alone^11,12^. The cell wall density^13^ is 1.5 g/cm${}^3$, and the UF resin’s$^{?}$ is around 1.3 g/cm${}^3$, so it could be possible to distinguish them with $\mu$CT. However, the relatively fine spreading of the UF resin, and its penetration into the wood cell wall only marginally affect the total density of the wood cell wall. This makes it almost impossible to discern the resin from the wood cell wall in $\mu$CT scans of MDF, to the best of our knowledge, even at high resolutions. Note that the 3D imaging of adhesive resins with $\mu$CT is already challenging in WBPs such as plywood, in which local glue layers can be found. Previous investigations of such panels have required the addition of contrast agents for higher X-ray attenuation^14,15^. To study MDF, where the resin and wood fibers are heavily interlinked and where no coherent structure could help identify either phase from the other, contrast agents for X-ray attenuation should be used in large quantities, and may still not be sufficient: a method for unambiguous identification is required.

Exploiting an element’s K-edge^16^, dual-energy CT (DECT) was designed to identify phases, based on their elemental composition, with X-ray CT^10^. K-edges are discontinuities in an atom’s mass attenuation coefficient profile for X-rays as a function of their energy, exhibiting a strong increase when exceeding a threshold energy (the so-called K-edge). Using two scanning energies with a monochromatic X-ray source (at a synchrotron) allows K-edge imaging, i.e. elemental identification. However, synchrotrons are not readily accessible unlike lab-based X-ray scanners, which generally have polychromatic X-ray sources. With those sources, some enhancements can still be achieved with DECT, which are already of major interest for the medical industry^17,18^. Nevertheless for finer material investigations on wood, DECT has so far been inconclusive, e.g. to evaluate the moisture content in a WBP^19^.

Building on the extensive knowledge of the polychromatic properties of our X-ray CT scanners^20^, a tailored lab-scale DECT methodology was designed. Its purpose was to investigate, if feasible, the 3D resin distribution inside a MDF panel, in which the resin was doped with a contrast agent carefully selected for its K-edge energy. To corroborate the DECT signal, elemental mapping of the MDF surface was achieved with scanning electron microscopy and energy-dispersive X-ray spectroscopy (SEM-EDX). This work presents a first glimpse of the UF resin distribution inside MDF. To the best of our knowledge, it constitutes the only complete characterization of this distribution in 3D.

## Results

**Figure 1:**
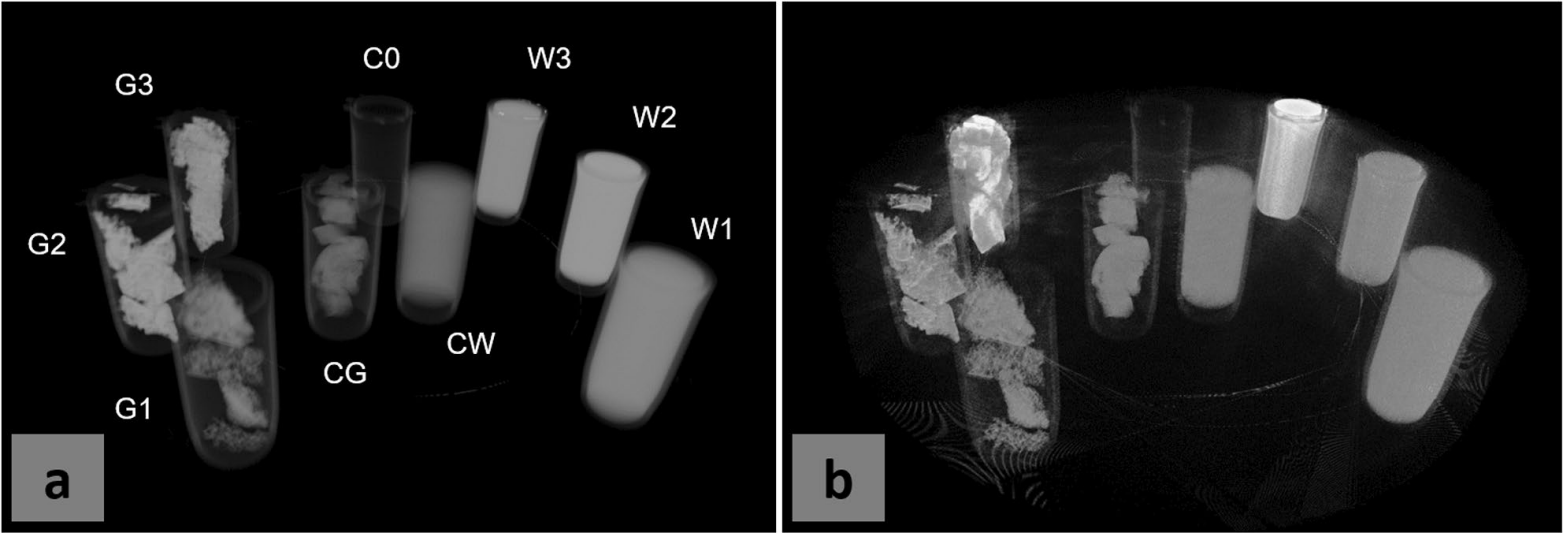
Greyvalue reconstructions obtained with standard absorption CT or DECT, at different contrast agent concentrations. For KBr-doped samples, DECT markedly increases the brightness achieved otherwise. Water-based samples W1: H${}_2$0 + 2 wt% KI; W2: H${}_2$0 + 10 wt% KI; W3: H${}_2$0 + 20 wt% KBr; Glue samples G1: UF + 2 wt% KI; G2: UF + 10 wt% KI; G3: UF + 20 wt% KBr; Control samples C0: empty tube; CW: H${}_2$0; CG: UF resin. (**a**) standard absorption CT image of a collection of Eppendorf tubes, filled with different solutions, (**b**) DECT image of the same sample collection. Notice how the Bromide-filled tubes stand out in the DECT image.

The feasibility of identifying doped UF resins with absorption $\mu$CT or DECT was investigated before making MDF panels containing doped resin. Nine Eppendorf tubes were prepared, three of which were filled with an aqueous solution containing water and a contrast agent (W1: H${}_2$0 + 2 wt% KI; W2: H${}_2$0 + 10 wt% KI; W3: H${}_2$0 + 20 wt% KBr). Three tubes were filled with UF resin samples (G1: UF + 2 wt% KI; G2: UF + 10 wt% KI; G3: UF + 20 wt% KBr), the same resins that would be used for making the experimental, stained panels. Finally, three tubes were control tubes: one was empty (C0), one contained distilled water (CW), and one contained pure UF resin (CG). In Fig. 1a, the reconstruction of a $\mu$CT scan (isotropic voxel size was 50 $\upmu$m) of these tubes is shown. At sufficient concentrations, the contrast agents might allow to differentiate the doped glue samples from the pure glue sample in absorption tomography. However, even at 20 wt% KBr (sample G3), the contrast is relatively small and thus the segmentation non-trivial. Conversely, the DECT image of the same field of view, shown in Fig. 1b, shows a marked contrast between the KBr-doped samples and the control samples, and allows for certain segmentation despite the higher noise levels. Extracting the ratio $r_i$ of the mean greyvalue in samples W${}_1$, W${}_2$, and W${}_3$, divided by the mean greyvalue in the control CW, results in $r^{\text {abs}}_1 = 1.93 \pm 0.09$, $r^{\text {abs}}_2 = 3.70 \pm 0.14$, $r^{\text {abs}}_3 = 2.49 \pm 0.11$, respectively, for the standard absorption reconstruction. In the DECT image instead, these ratios are $r^{\text {DECT}}_1 = 1.07 \pm 0.21$, $r^{\text {DECT}}_2 = 0.93 \pm 0.30$, $r^{\text {DECT}}_3 = 1.94 \pm 0.17$: the relative greyvalue of voxels containing KBr has increased, while the greyvalue of any other voxel has decreased, sometimes below the noise levels.

**Figure 2:**
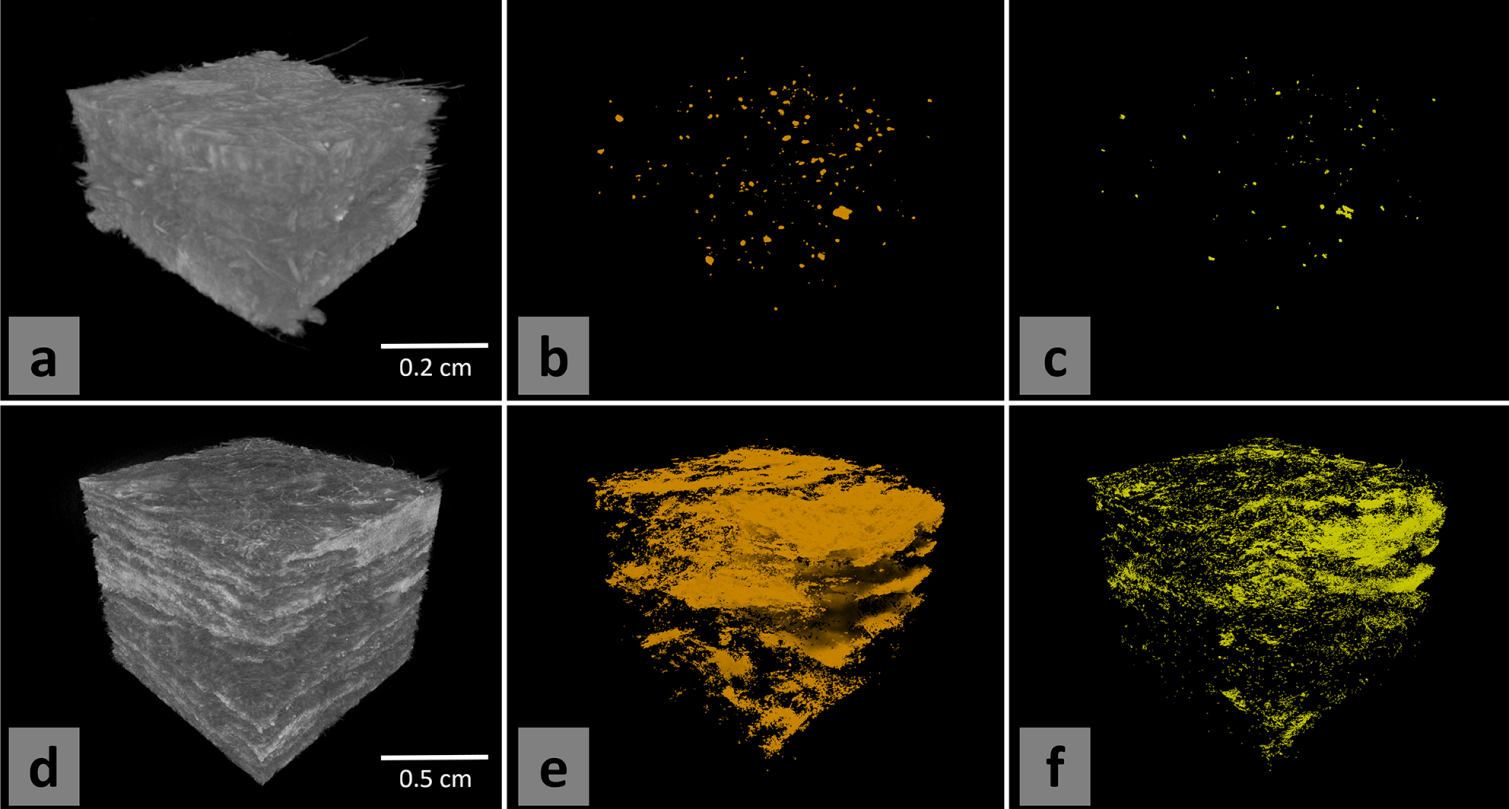
DECT in-situ visualization of the UF resin in experimental MDF samples, compared to low-precision standard absorption tomography. (**a**) control MDF sample, (**b**) segmented high-intensity voxels in the absorption image, (**c**) segmented high-intensity voxels in the DECT image. (**d**) KBr-doped experimental MDF sample, (**e**) segmented high-intensity voxels in the absorption image, (**f**) segmented high-intensity voxels in the DECT image.

Success of the feasibility experiment led to the making of a control MDF panel and of a prototype MDF panel, in which the UF resin is mixed with 20 wt% KBr. In such doped-resin samples and as far as our methods permitted to observe, the contrast agent was finely and evenly distributed in the UF resin. Small MDF samples were cut for $\mu$CT scanning (Fig. 2a,d, respectively). As seen in Fig. 2b,e, standard absorption X-ray CT imaging resulted in imprecise, because bulky, segmentation. The threshold was set at the end of the dynamic range of voxel greyvalues, so that only the tail of the greyvalue distribution is segmented. As seen in "Methods" section, the reconstructions were made so that the histogram of grey values and the numerical threshold chosen were qualitatively comparable. In other words, both in the absorption and DECT images, the segmented voxels correspond to those voxels that cannot belong to the same phase as the bulk of the material. In the absorption image, these extra-ordinary voxels may correspond to high-density impurities in the material, $\mu$CT artefacts (in particular phase contrast), or UF resin spots (presence already inferred from the presence of bromide). Segmenting these high-intensity voxels in the scan of a control sample (without KBr) results in a significant amount of points that could be UF resin (Fig. 2b), but cannot be identified with certainty. In contrast, there are almost no high-intensity voxels in the DECT image of the same control sample (Fig. 2c), which is expected for a control sample. DECT only significantly highlights voxels containing a sufficient amount of relatively heavy elements, which can be impurities in wood, or residues from cutting and grinding it into fibers. On the KBr-doped sample however, glue spots are visible both in the absorption (Fig. 2e) and the DECT image (Fig. 2f). Yet, DECT helps identifying with improved reliability the presence of bromide, as the segmented phase shows less voxels than in the absorption image and appears refined.

**Figure 3:**
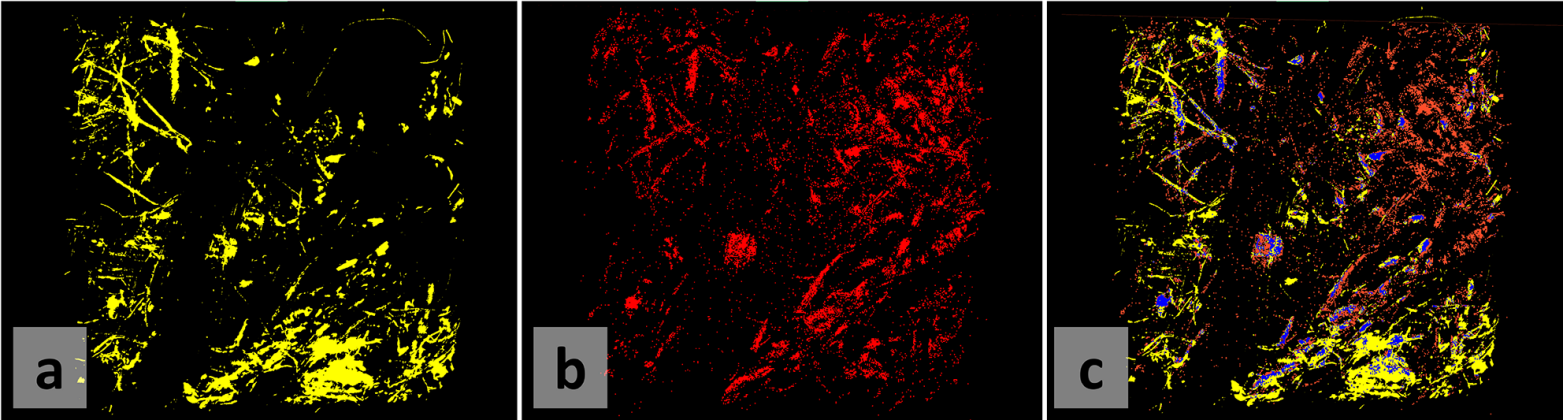
SEM-EDX validation of DECT on the surface of the sample, despite imaging differences making the overlay imperfect. (**a**) segmented high attenuation voxels in the DECT image, (**b**) segmented bromide mapping from SEM-EDX, (**c**) overlay of the SEM-EDX elemental mapping and the DECT image after automated registration. The blue phase indicates exact overlay of the two images: about 1/3 of the SEM-EDX segmented pixels.

**Figure 4:**
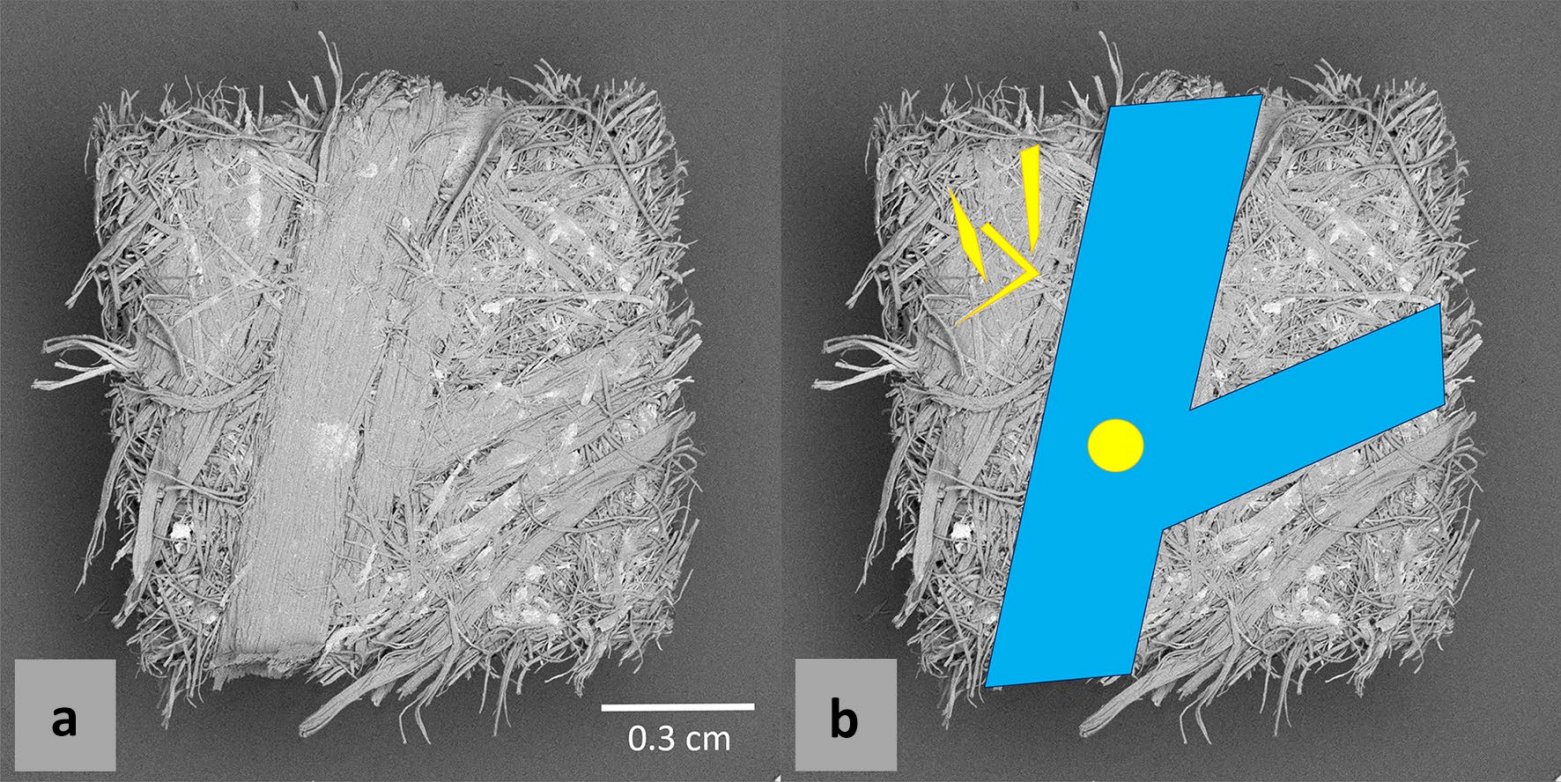
Scanning electron microscopy image of the sample’s surface, and primary feature schematics. (**a**) Original SEM image; (**b**) SEM image overlaid with the shapes of easily-identifiable features: in blue, two fiber bundles intersecting; in yellow, some important glue spots that can be related to Fig. 3.

To validate that DECT enables the imaging of KBr-doped glue, the SEM-EDX mapping of bromide on the top surface of the KBr-doped sample is shown in Fig. 3a. Next to it (Fig. 3b), the segmentation of high-intensity voxels in a top slab of the $\mu$CT scan of the same sample shows corresponding patterns, which are furthermore schematized in Fig. 4: a resin blob at the center, and UF-saturated fibers that are recognizable from one image to the other. Moreover, the rough amount of identified resin pixels between DECT and SEM-EDX (see Fig. 3) match, something that would not be the case with standard absorption tomography: as seen in Fig. 2b, standard absorption tomography results in a bulky segmentation when segmenting only the brightest voxels. The automatic registration of the images from the two imaging modalities, shown in Fig. 3c, quickly converged to a convincing solution. The overlay is not perfect, with some features being incompletely matching, but is coherent nonetheless. Note that the information cannot be comparable everywhere in the image: SEM-EDX encodes information about the topological surface of the sample, with every pixel potentially having a different height in space. Instead, DECT reconstructs information about the sample in 3D, and the image retrieved in Fig. 3 corresponds to the trend observed within a fixed volume in space. This entails that some glue as seen in the DECT image can be below the surface visible to SEM-EDX, and vice-versa. However overall, the K-edge-based elemental identification (SEM-EDX) and the energy range enhancement imaging (DECT) match, and allow to validate the DECT methodology for KBr-stained glue imaging.

## Discussion

The DECT methodology that was developed for this study does not allow “pure” monochromatic single element K-edge imaging. Instead, it can emphasize the grey value of voxels containing relatively heavy elements, and more specifically those elements with a K-edge in the range 12–29 keV (see "Methods" section; Fig. 6). Those are the elements with atomic number between 33 (arsenic) and 50 (tin). Iodine, with K-edge around 33 keV, is therefore just out of this energy range, and its contrast enhancement with DECT is less than that of bromide with K-edge around 13 keV (Fig. 1). While formal elemental identification cannot be conducted with the current methodology as it would require monochromatic X-ray sources^21^, we hypothesize that bromide is the only relatively heavy element present in the MDF. Wood-based products such as MDF are indeed composed of light elements (typically C, H, O, and some N), and a high DECT signal (i.e. a high linear attenuation coefficient value difference between the two images required for DECT) is expected to correspond almost exclusively to voxels containing bromide. We hypothesize that the presence of bromide is a good indicator of the presence of the UF resin, because exploration with $\mu$CT and SEM showed no evidence of dissociation between the resin and bromide before or after heat-pressing and drying. Furthermore, KBr has a relatively high solubility (678 g/L in water at 25 ${}^\circ$C), and was therefore expected to be well mixed in the UF resin, even at 20 wt%. However, DECT in that case still faces two major limitations: the partial volume effect (PVE), and phase mixing. The $\mu$CT images are indeed rasterized, meaning that the data exists in voxel space and within a voxel, with a pitch of 10 $\upmu$m, the DECT signal is averaged. Therefore, the DECT signal only significantly highlights (and allows to numerically threshold) those voxels which contain a sufficient amount of bromide compared to their surroundings. The detectability is a complex function of a number of acquisition parameters, and a detection limit cannot be precisely determined. Furthermore, phase mixing corresponds to the mingling between the glue phase and the wood fiber phase, i.e. the partial penetration of the wood cell wall by UF resin. It is a feature that is considered positive for the strength of the MDF panel, as partial penetration of the resin into the wood fibers allows anchoring and bonding to the surrounding fibers^4^. However, we can then expect the wood fiber structure to contain some amount of KBr-doped resin, depending on which exact fiber/part of fiber is being investigated. As this relates to the PVE and phase mixing, the critical amount of UF resin saturation inside the wood cell wall to ensure highlighting in the DECT image is also currently unknown. Note that originally, conventional absorption tomography was expected to highlight the doped-resin sufficiently for segmentation in the material. However it was not, and DECT thus provided a way to sharply increase the contrast, and more importantly allow for precise resin identification. Despite the success and proof of concept of the experiment, some improvements could be envisaged. It is shown (see "Methods" section) that with the current methodology, the DECT contrast would be maximal for an element with its K-edge at 20 keV, i.e. molybdenum. While molybdenum is not suitable for UF resin-doping, the choice of material filters for DECT could instead be tweaked to maximize the DECT contrast at 13 keV, i.e. the K-edge of bromide. To do so, thinner Sn and Cu filters (see "Methods" section) could eventually be used, although thinning the material filters would also result in lowering the DECT signal. In that case, a different strategy would be followed. The search for balance between the different constraints inherent to lab-based DECT requires extensive use of tomography simulation software, such as Arion^20^, to find potentially suitable scanning parameters.

As seen on the surface of the sample with both SEM-EDX and DECT (Fig. 3), the mapping of bromide leaves two thicker lines relatively void of identified glue spots (it forms an empty V-shape across the images). Inspection of the surface with SEM (Fig. 4) shows that these lines correspond to two fiber bundles intersecting. The presence of fiber bundles in MDF panels is of interest, and particularly for their relation to the UF resin. Automatic segmentation of these bundles in $\mu$CT scans has been achieved^22^, but requires higher spatial resolution to work reliably. In the present $\mu$CT scan at 4 times lower resolution, fiber bundle identification is more difficult. It is expected however, that the UF-resin would scarcely penetrate fiber bundles, since during the manufacturing of MDF, the moisture content of the wood fibers has to be increased for better (and sufficient) resin penetration. However, this requires steam-cooking the wood fibers, a process that is relatively expensive. The manufacturers are therefore generally not able to reach optimal moisture contents in the bulk of the wood fiber resource (i.e. single wood fibers)^5^, and are thus even less likely to reach high moisture content (which conditions the resin content) in the core of the fiber bundles.

Many factors can have an impact on glue distribution: resin curing rate, catalyst content, methods of fiber refining, wood species-specific chemical characteristics, fiber treatment or even the hot pressing process^5^. The KBr-doped panel investigated in this study is the product of a third manufacturing experiment (with a laboratory-scale press), following major improvements at each iteration. In the KBr-doped panel, the real issue however, are the resin blobs which are neither expected, nor realistic. Upon visual inspection of the panels’ cross section some blobs can be identified, which are identified as resin blobs based on the absence of porosity, which is inherent to wood and wood fibers. These visually-striking blobs do not appear in commercial-grade MDF panels. Note that MDF is a particularly challenging material to reproduce accurately at the laboratory scale. The industrial production of MDF requires line presses operating at several MPa, over tens of meters^23^. Nevertheless, additional efforts could be made to investigate commercial-grade MDF free of contrast agent. Specifically, the presence of UF-resin coincides with the presence of nitrogen^9^. In this study, SEM-EDX identification of nitrogen was tried on a sample from a commercial (non-experimental; with finer glue distribution) panel. Unfortunately, SEM-EDX is less sensitive to light elements, and nitrogen presence is low (6.30 ± 27.97 wt% in the field of view considered then). These factors made the inspection of the difference in resin penetration in fibers or fiber bundles inconclusive. More experiments can still be attempted to answer how far UF resin penetrates into the wood fiber structures, for instance with other imaging techniques (e.g. confocal laser scanning microscopy^9^).

Finally, it should be mentioned that one of the major practical issues in the DECT methodology is sample motion (sub-pixel motions increasing uncertainty in DECT) and deformation. Although the acquisition protocol can be designed such that there are no movements of the sample stage other than rotation, the volumes do not align perfectly at high resolutions (voxel size 10 $\upmu$m and below). The small misalignment effects can be due for instance to insufficient sample conditioning to the relative humidity of the scanner room, or to spot shift (see "Methods" section) between the two scans. For these reasons and others, image registration is necessary to perform DECT at high resolutions.

## Methods

### Arion simulation software

**Figure 5:**
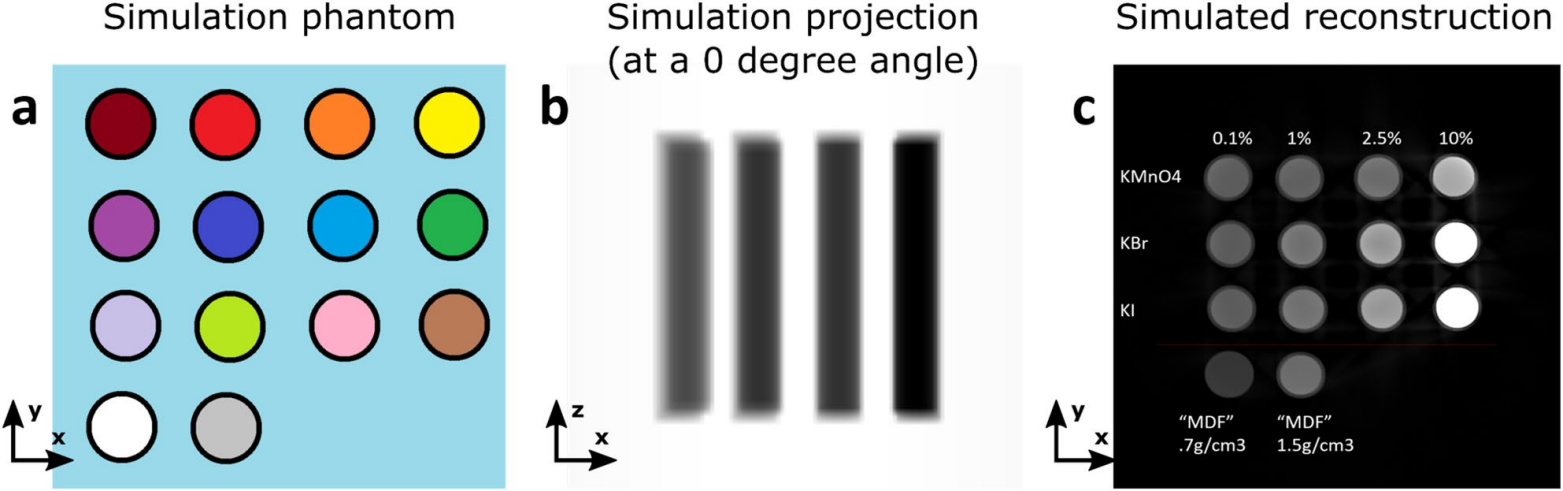
Arion simulation framework of X-ray projections (**b**) of a phantom (**a**) consisting of an array of Eppendorf tubes (the phantom is panned in the vertical direction). From the simulated projections at different angles, CT reconstruction (**c**) can be done to examine the expected X-ray attenuation contrast between different solutions.

The radiography simulator Arion^20^, developed at the Ghent University Centre for X-ray Tomography (UGCT, https://www.ugct.ugent.be), was used throughout this study. It condenses the in-depth knowledge of the different systems built at UGCT, especially the polychromatic properties of the X-ray tube and detector response. Given a user-drawn phantom, such as the one presented in Fig. 5, and the scanning parameters and geometries, accurate X-ray radiographies (or projections) can be simulated^20^. From these projections, any CT reconstruction framework can be used to retrieve the 3D image of the phantom as seen through the CT scanner. In this study, Arion enables the testing of different contrast agents and concentrations before preparing physical samples. Three relatively affordable potassium-based salts (KMnO${}_4$, KBr, and KI) were initially selected, considering that a large-scale industrial test was envisioned requiring considerable amounts of contrast agent eventually. Together, these salts present a variety of absorption K-edges in the range of $\sim$6 to $\sim$33 KeV, which allows some flexibility in testing. With the simulation of absorption CT reconstructions, the possibility of perfectly segmenting (i.e. separating), with manual thresholding, a given salt solution from an ideal “MDF solution” was evaluated. This experiment enabled us to exclude KMnO${}_4$ from further testing, and to restrict the manufacture of KBr- or KI-doped samples to only a couple possible concentrations. Note that the MDF solution against which thresholding was evaluated is not real; it is an imaginary solution with the same elemental composition and density as the commercial MDF panels investigated otherwise. The elemental composition of MDF was determined with mass spectrometry on two MDF samples, using a FLASH 2000 organic elemental analyzer (Thermo Fisher Scientific, Waltham, USA) equipped with a thermal conductivity detector. The composition of MDF was found as 54.81% C, 37.84% O, 6.74% H, and 0.61% N on average.

These in-silico trials were confirmed with a real scan of Eppendorf tubes filled with different contrast agent solutions, as presented on Fig. 1. Note that since the wood fiber solution is not real, it was approximated with a tube filled with distilled water (whose electron density is relatively close to that of the wood cell walls). If a solution achieves good contrast with water, it is expected to also reach sufficient contrast with the wood cell walls.

### Doped-resin MDF samples

Commercial MDF contains standard urea-formaldehyde (UF) resin, which is similar in density and chemical composition to wood fibers. As such, it is not identifiable in-situ with X-ray $\mu$CT. Collaboration with the Belgian MDF manufacturer (Unilin, https://www.unilin.com/en) allowed the production of custom MDF panels, in which contrast agents were mixed in the adhesive resin prior to panel forming. These panels were realized in the R &D department of Unilin, where the main difference with the production line is the spraying of resin onto the fibers. Raw wood fibers (*Picea abies*) were otherwise extracted from the main production line, and are thus identical to those found in commercial panels. A dry process was used to obtain MDF panels at a final density of 650 ± 50 kg/m${}^3$. Potassium-based salts (KBr and KI) were mixed in the UF resin (identical to that used in the main production line), at 10, and 20 wt%. After initial tests, which eliminated KI for resin identification with DECT, only the KBr 20 wt% panel was retained. In addition, control panels were produced, in which no contrast agent was added to the UF resin. No hardener was used. The resin, dosed at 12 % for dry resin on dry fibers, was sprayed with an airless spraying device (TriTech Industries, USA) with a nozzle (Schlick, Germany) set with a 60$^\circ$ angle.

### Dual-energy computed tomography (DECT)

**Figure 6:**
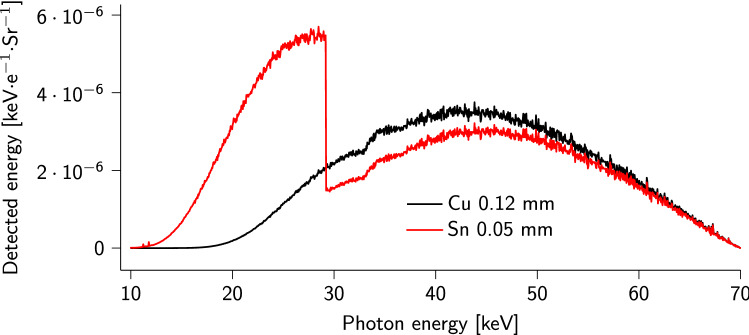
Detected energy for the specific tube-detector combination used at Nanowood, modulated by the theoretical elemental absorption profiles of the Cu and Sn filters used in this study. The difference between these two curves gives an insight on the energy ranges that would be highlighted by this particular DECT setup.

The samples were scanned with the $\mu$CT system Nanowood^24^, built at UGCT. For each scan, 2001 projections were acquired over a 360$^\circ$ rotation. The tube was set at a voltage of 70 kV, power of 16 W, and the detector’s exposure was set at 1.25 s. Two material filters were used subsequently, whose materials (Sn and Cu) and thicknesses (0.05 mm and 0.12 mm, respectively) were chosen based on simulations. The Setup Optimizer module of Arion was used to predict the energy deposited in the X-ray detector when the X-ray spectrum was first filtered by a material filter of a given thickness. The two adjustable parameters were the material of the filter, and its thickness. Several filter combinations were tried, with the goal of identifying two filters that would lead to a maximal difference in detected energy at the lower ranges of the X-ray spectrum. As seen in Fig. 6, the detected energy from the Sn-filtered spectrum, i.e. the product of the filtered X-ray spectrum and the detector response as a function of X-ray energy, shows a discontinuity at the K-edge of Sn (29 keV). In effect, filtering with a Sn filter reduces the detected signal for elements with K-edge above 29 keV. On the other hand, Cu having its K-edge around 9 keV results in no visibility changes from mid to high energies; the filter’s effect being rather to remove the low-energy photons. The standard absorption tomography scan was done with the Cu filter to prevent beam hardening in the sample. For DECT overall, the difference between the two detected spectra shown in Fig. 6 could highlight elements with K-edges in the range 12–29 keV. Integrating the detected energy profiles shows that X-rays with energies below 29 keV contribute 40 % to the Sn-filtered signal, and only 10 % to the Cu-filtered signal. However, because the sharp increase in X-ray absorption at an element’s K-edge decreases slowly above the K-edge energy, the difference between the two spectra is better exploited for those elements whose K-edge and tail are in the range 12–29 keV, instead of elements with K-edge energy too close to 29 keV. While a precise definition of the optimal energy range is out of the scope of this research, we can assume the current setup to work best for elements with a K-edge energy between 12 and 20 keV. Of all elements present in the samples considered here, only Br has its K-edge in this range, hence the highlighting of only those samples containing KBr in Fig. 1b.

By using the tube at the same voltage for the two images, the location of the spot inside the X-ray tube, out of which the X-ray beam emanates, is preserved. This eventually allows for easier registration before computing the difference between the two images. While image registration can be accurate, this study’s high spatial resolution combined with the hygroscopic properties of wood fibers (whose environment affect the dimensions), require extra measures to limit sample movement and deformation. The structure of interest is indeed the wood fiber, whose small dimensions amplify the relative importance that sub-pixel motions at the edges could have. For this reason, every precaution was taken to avoid sample motion, although image registration was nevertheless necessary. However, the fixed-voltage strategy limits the optimum spectra choice somewhat, since changing the tube voltage would allow to obtain more suited X-ray spectra for DECT purposes. Here, one scan was acquired with the Cu filter, and a second scan was acquired immediately afterwards with the Sn filter instead, without moving the sample.

The 16-bit volume reconstructions were obtained with Octopus Reconstruction^25^. The Paganin phase retrieval algorithm^26^ was used, with linear attenuation parameter $\mu = 0.2$ $\text {cm}^{-1}$ (close to wood), and $\delta = 10^{-5}$. In these reconstructed images, the approximate voxel pitch was 10 $\upmu$m. Importantly, the dynamic range of the reconstructed images was set to fix numerical boundaries across the scans. Since the scans were acquired with the same scanning parameters otherwise, this ensures that the scans are quantitatively comparable before DECT. Moreover, setting these boundaries conservatively allows to prevent saturation in the image resulting from image subtraction. Volumes were registered in Dragonfly 2020.1 (Object Research Systems, Montreal, Canada—free of charge for non-commercial use), after which they were subtracted in Fiji^27^ as Sn–Cu. The KBr-doped sample scanned with DECT was then prepared for scanning electron microscopy. The bottom was trimmed, as taller samples can cause problems for SEM, which only analyzes the top surface.

### Scanning electron microscopy and energy-dispersive spectroscopy (SEM-EDX)

SEM was conducted at the Department of Geology, Ghent University, with the MIRA3 scanning electron microscope (TESCAN, Brno, Czech Republic) equipped with a field emission gun and an energy-dispersive spectroscopy detector (EDAX element 30). The working distance was set to around 15 mm, the optimal working distance for this specific device. Prior to imaging, the sample was coated under vacuum with a thin layer of carbon to minimize charging effects in the image. It was then imaged with back-scattered electrons (BSE), which enables contrast based on chemistry rather than topography (as is the case in secondary electron mode). Image acquisition was performed using the Mira-TC software provided by Tescan (Brno, Czech Republic). The acceleration voltage was set to 15.0 kV and the spot size was 98.6 nm. Images were acquired at a magnification of 83 times, giving a field of view of 6.65 mm, with an isotropic pixel size of 8.52 $\upmu$m.

Energy-dispersive spectroscopy (EDX) was performed using the EDS detector and the APEX software, which allows to collect spectra at either points, lines or areas on the sample. Chemical mapping was performed, allowing to create a map of bromide on the surface.

### Data fusion and DECT validation

SEM-EDX provides a 2D image of the sample’s surface, while DECT results in a 3D image. To compare the two datasets, the DECT volume was first rotated in Dragonfly, so that the surface would be aligned with a single slice. Then, a mean intensity slab of 30 slices (representing a thickness of 300 $\upmu$m) was taken at the top of the sample, and manually thresholded to isolate the high-intensity voxels. It was then registered in Dragonfly, with the SEM-EDX mapping of Bromide.

## Data Availability

The datasets used and/or analysed during the current study are available from the corresponding author upon request.
